# Cardiovascular Disease Risk and Outcomes in Patients Infected With SARS-CoV-2

**DOI:** 10.7759/cureus.12783

**Published:** 2021-01-19

**Authors:** Diana Espinoza, Manasa Jasti, Umme Haani Malwi, Christine Junia

**Affiliations:** 1 Internal Medicine, MacNeal Hospital, Berwyn, USA

**Keywords:** covid-19, cardiac compromise, risk factors, troponin, myocarditis, inflammation, chest pain, cardiovascular, syncope, mortality

## Abstract

Cardiovascular involvement is one of the end-organ complications commonly reported in coronavirus disease 2019 (COVID-19). It has also been postulated to be an independent risk factor for increased mortality in COVID-19-infected patients. With such a significant effect of COVID-19 on the cardiovascular system and vice versa, it is pivotal for physicians to observe this association closely for improving management and understanding prognosis in these patients. Here, we present three patients and describe their baseline cardiac risk factors, the cardiac complications they developed in association with COVID-19 infection, and their varying outcomes.

## Introduction

Coronavirus disease 2019 (COVID-19) has spread worldwide, and with significant associated mortality, new questions about fatality predictors arise. Although the respiratory system is most commonly involved, literature shows that a wide range of cardiovascular complications are associated with poor outcomes, especially in critically ill patients [1]. Baseline cardiovascular disease (CVD) is a risk factor for COVID-19, playing a direct role in predicting increased morbidity and mortality [2]. The mechanism of cardiac injury could be related to either direct injury through angiotensin-converting enzyme 2 (ACE2) receptors, which are widely distributed in the lungs and heart, or could be secondary to demanding ischemia or as a result of multisystemic inflammatory response [3]. Cardiovascular involvement can present as acute myocardial injury, heart failure, myocarditis, and lethal arrhythmias, some of which are described in our case series [3].

## Case presentation

Case 1

**Figure 1:**
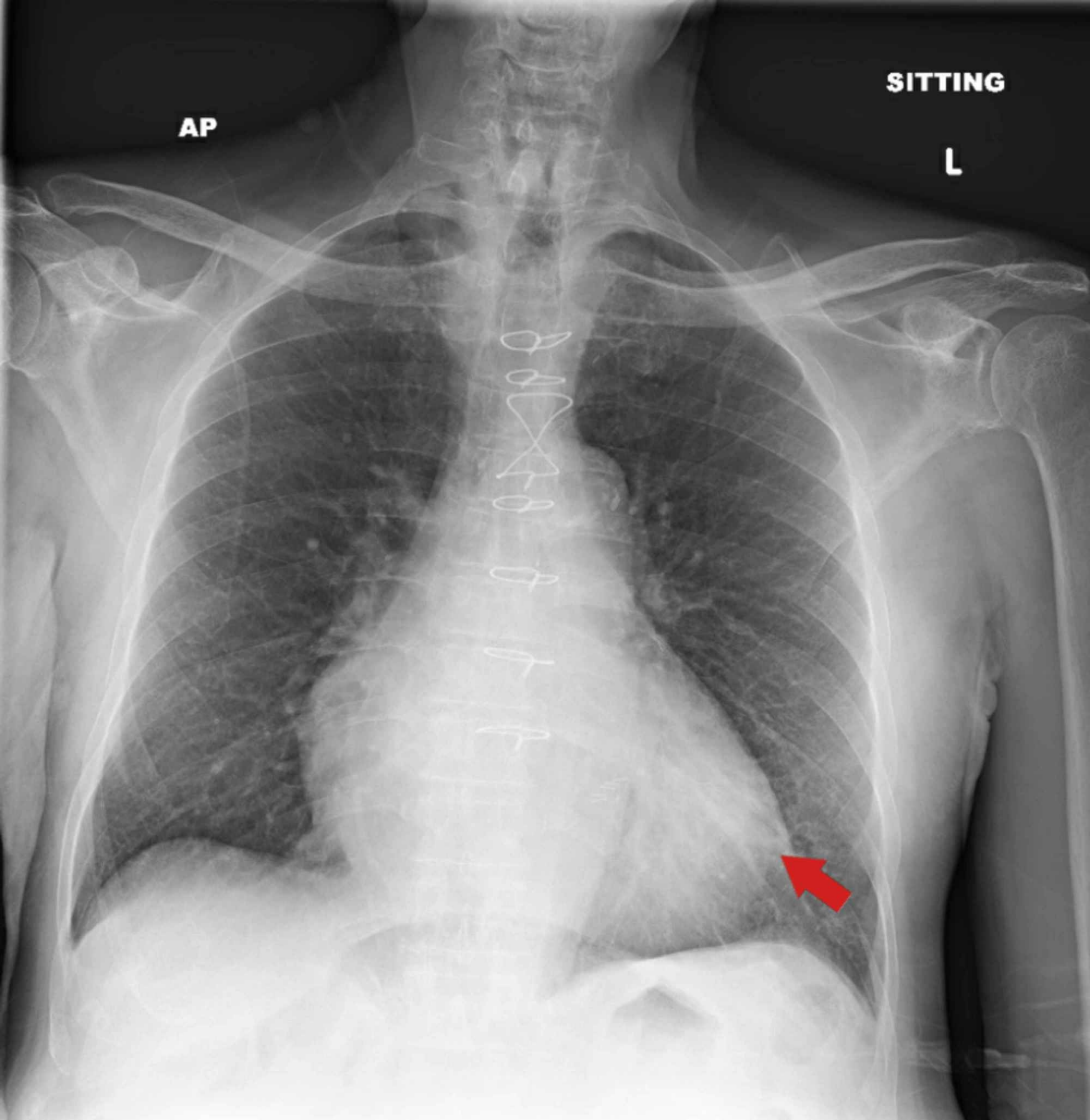
CXR on admission showed moderate cardiomegaly and no lobar consolidation. CXR, chest X-ray

**Figure 2:**
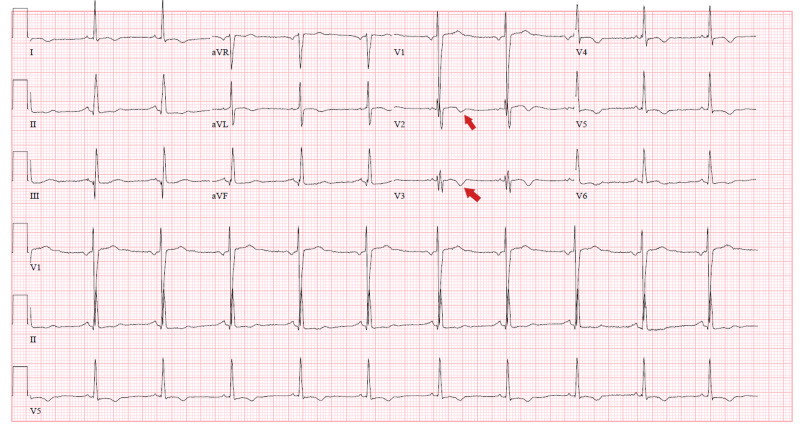
ECG on admission showed nonspecific T-wave abnormalities in anteroseptal leads (red arrows). ECG, electrocardiogram

An 80-year-old male with a past medical history (PMH) of coronary artery disease (CAD), ischemic cardiomyopathy (ICM) with ejection fraction (EF) of 30%, atrial fibrillation, hypertension (HTN), and type 2 diabetes mellitus (T2DM) was admitted for presyncope. He denied chest pain, dyspnea, cough, or fever. Vital signs and physical examination were unremarkable. Chest X-ray (CXR) showed moderate cardiomegaly and no lobar consolidation (Figure 1). Electrocardiogram (ECG) showed nonspecific T-wave abnormalities in anteroseptal leads (Figure 2) with initial troponin of 0.63 ng/mL (Tables 1, 2). Transthoracic echocardiogram (TTE) showed a drop in EF to 20% from the baseline with new anteroseptal and apical akinesis. He developed fever during his hospitalization and eventually tested positive for COVID-19. He also had an episode of nonsustained ventricular tachycardia during the admission. The patient was discharged home with outpatient cardiac workup.

Case 2

**Figure 3:**
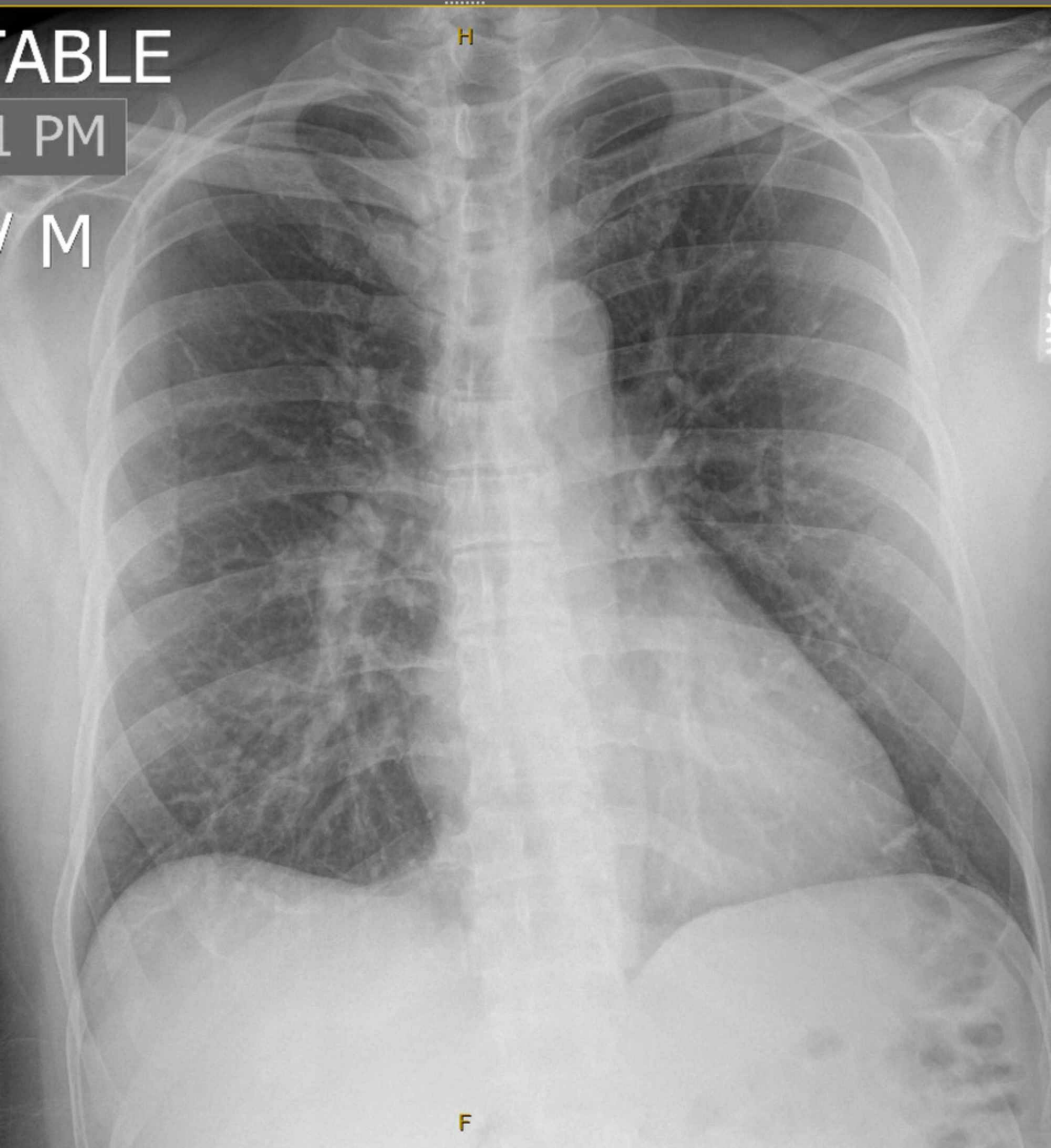
CXR on admission showed no acute cardiopulmonary process. CXR, chest X-ray

**Figure 4:**
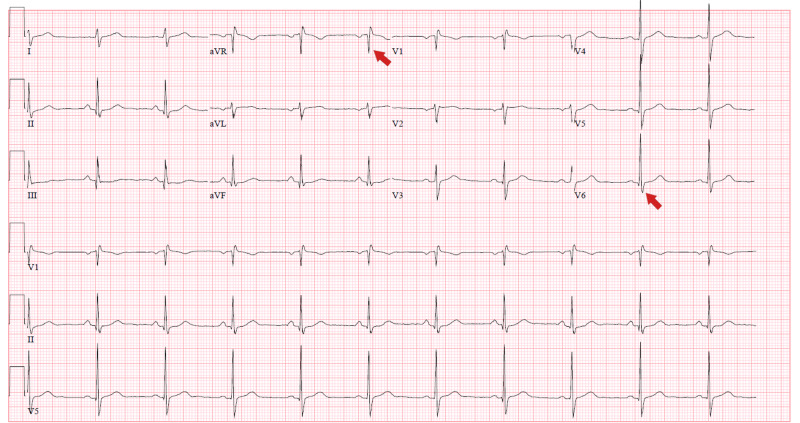
ECG on admission showed sinus rhythm with incomplete RBBB (red arrows). ECG, electrocardiogram; RBBB, right bundle branch block

**Figure 5:**
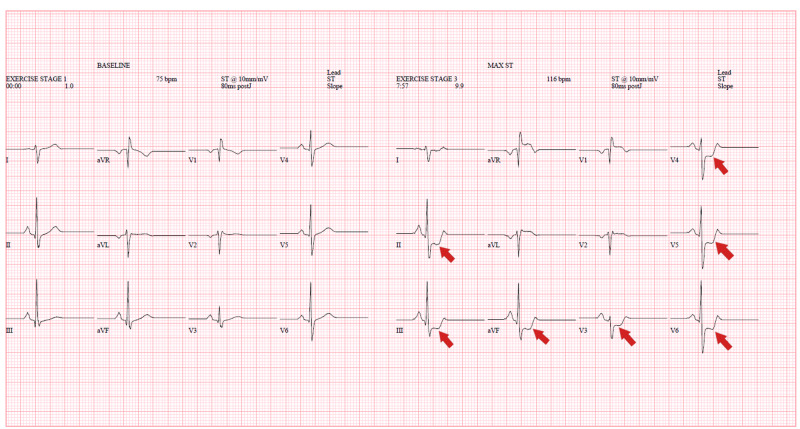
Stress ECG showed 2-mm ST-depression in infero-lateral leads (red arrows), consistent with exercise-induced ischemia. ECG, electrocardiogram

A 54-year-old male with PMH of hyperlipidemia (HLD) presented with a near syncopal episode. He denied any other symptoms. Vital signs, physical examination, and CXR were unremarkable (Figure 3). ECG showed an incomplete right bundle branch block (RBBB) (Figure 4) with an initial troponin of 0.55 ng/mL, which peaked at 0.61 ng/mL (Tables 1, 2). During his hospitalization, he developed a transient episode of sinus bradycardia 40 beats per minute and hypotension to 90/50 mmHg. He developed fever and eventually tested positive for COVID-19. Stress ECG revealed normal resting wall motion with normal global systolic function and showed abnormal 2-mm ST-depression in infero-lateral leads (Figure 5). The patient was discharged home and planned further workup as an outpatient.

Case 3

**Figure 6:**
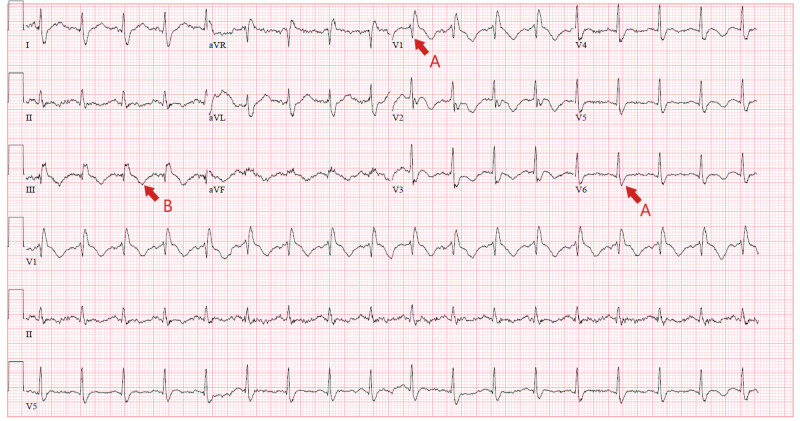
ECG on admission showed sinus tachycardia, RBBB (red arrows A), T-wave abnormality on infero-lateral leads (red arrow B). ECG, electrocardiogram; RBBB, right bundle branch block

**Figure 7:**
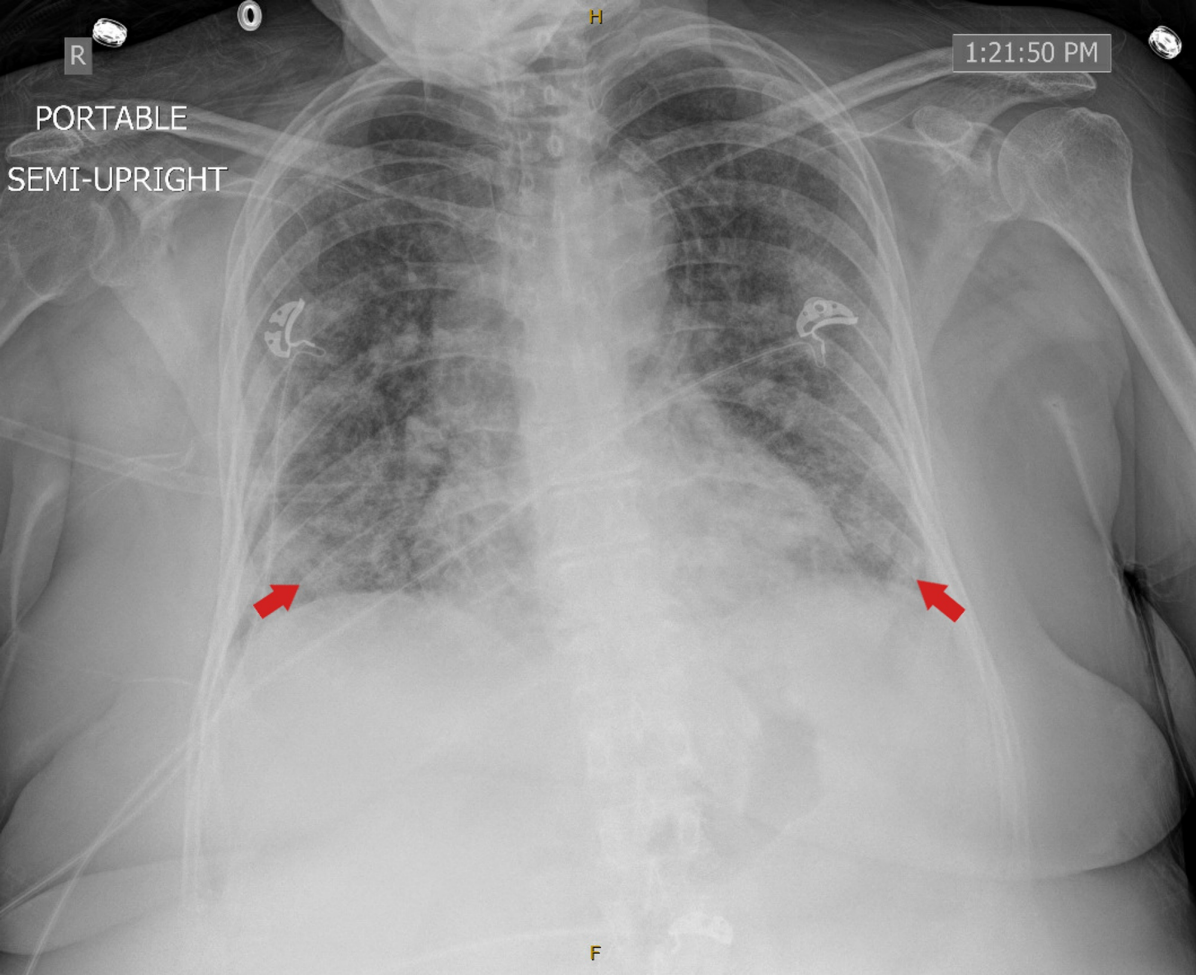
CXR on admission showed diffuse bilateral infiltrates. CXR, chest X-ray

**Figure 8:**
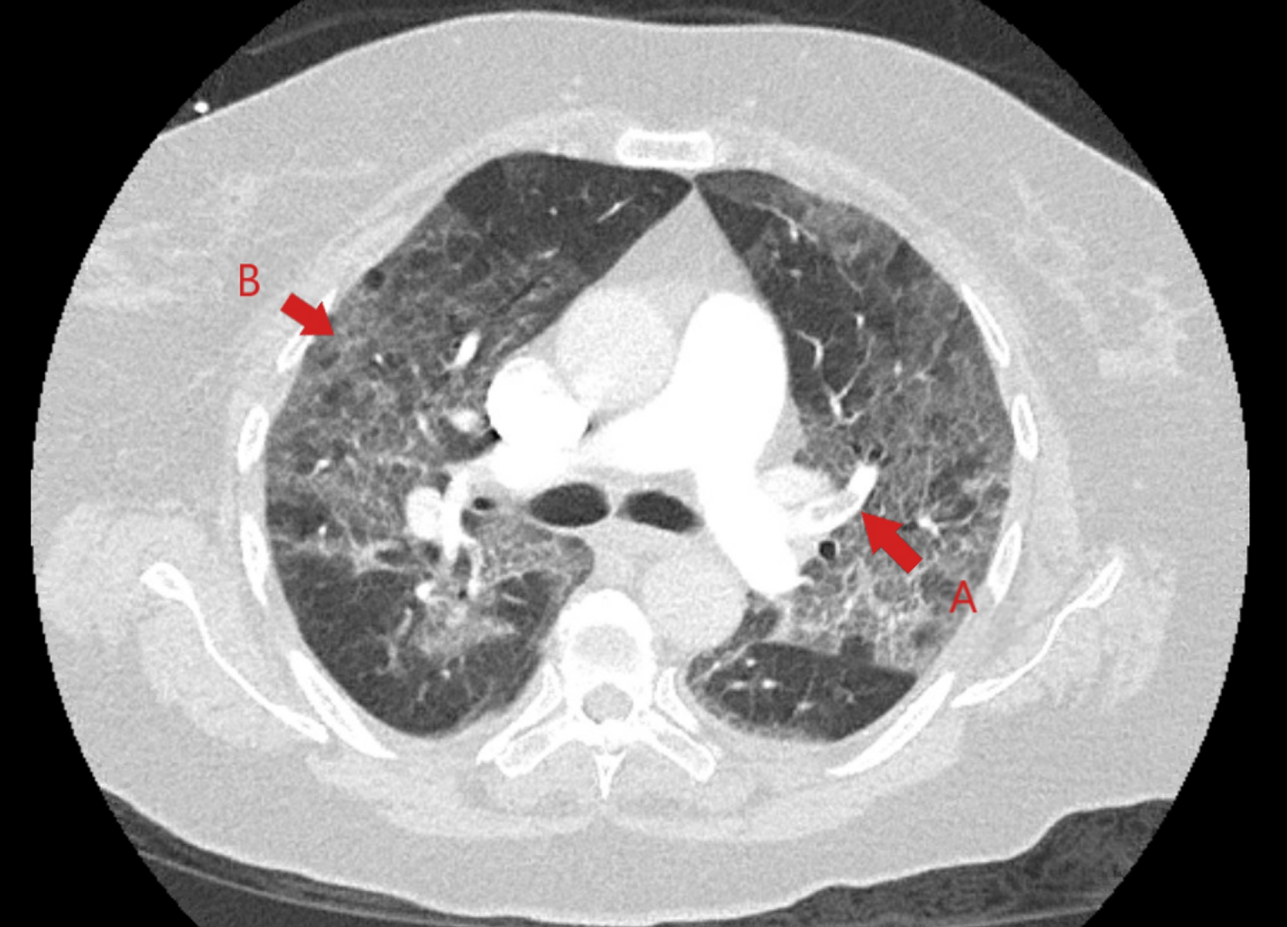
CT chest with IV contrast showed bilateral acute pulmonary thromboemboli (red arrow A) and diffuse bilateral multifocal ground glass opacities (red arrow B). CT, computed tomography

A 64-year-old female with PMH of hypothyroidism, who had tested positive for COVID-19 two weeks prior to admission, presented with altered mental status, dyspnea, and subjective fevers. She was febrile to 102.3 F, tachycardic to 115 beats per minute, tachypneic to 30 respirations per minute, saturating at 71% on room air, eventually requiring 6 L of oxygen supplementation via nasal cannula. Physical examination was unremarkable. ECG showed new RBBB (Figure 6) with an initial troponin of 3.63 ng/mL and peaked at 6.07 ng/mL (Tables 1, 2). CXR showed diffuse bilateral infiltrates (Figure 7). Computed tomography of the chest revealed acute bilateral small peripheral pulmonary emboli (Figure 8). Hospital course was complicated by acute hypoxic respiratory failure requiring intubation followed by cardiac arrest achieving return of spontaneous circulation (ROSC), septic shock requiring vasopressors, acute renal failure requiring hemodialysis, and methicillin-sensitive *Staphylococcus aureus* bacteremia. TTE showed normal left ventricular cavity size and normal global EF. Unfortunately, the patient expired.

 

**Table 1:** Clinical characteristics, diagnostic testing, and outcomes of the three cases. COVID-19, coronavirus disease 2019; CXR, chest X-ray; CAD, coronary artery disease; CABG, coronary artery bypass graft; ICM, ischemic cardiomyopathy; HFrEF, heart failure with reduced ejection fraction, HTN, hypertension; T2DM, type 2 diabetes mellitus; CVA, cerebrovascular accident; HLD, hyperlipidemia, RBBB, right bundle branch block

Variable	Case 1	Case 2	Case 3
Age (years)	80	54	64
Sex	Male	Male	Female
Presentation	Near syncope	Near syncope	Altered mental status and dyspnea
Respiratory symptoms	No	No	Yes
Time of symptom onset before presentation	One day	One day	One week
Timeline of COVID-19 testing	During admission	During admission	Two weeks before admission
CXR	Moderate cardiomegaly and no lobar consolidation	No acute cardiopulmonary process	Diffuse bilateral infiltrates
Cardiovascular risk factors	CAD status post CBBG, ICM, HFrEF, chronic atrial fibrillation, HTN, T2DM, CVA	HLD	Hypothyroidism
ECG	Nonspecific T-wave abnormalities in anteroseptal leads	Sinus rhythm with incomplete RBBB	Sinus tachycardia, RBBB, T-wave abnormality in infero-lateral leads
ECG	EF 20% with new wall motion abnormalities	Normal resting wall motion with normal global systolic function at rest and exercise	Normal left ventricular cavity size and global EF with no evidence of valvular vegetations.
Other cardiac workup	None	Stress test: 2-mm ST-depression in infero-lateral leads, consistent with exercise-induced ischemia	None
Complications	Nonsustained ventricular tachycardia	Transient bradycardia, hypotension	Cardiac arrest, bilateral pulmonary embolism, acute renal failure, septic shock
Mortality	No	No	Yes
Angiography	Outpatient	Outpatient	-

**Table 2:** Laboratory testing for three cases and reference ranges. BNP, brain natriuretic peptide; LDH, lactate dehydrogenase; CRP, C-reactive protein

Test	Case 1	Case 2	Case 3	Reference range
Troponin on admission	0.63 ng/mL	0.55 ng/mL	3.63 ng/mL	0.00-0.01 ng/mL
Troponin peak	0.63 ng/mL	0.61 ng/mL	6.07 ng/mL	0.00-0.01 ng/mL
BNP	-	-	599 pg/mL	1-100 pg/mL
Inflammatory markers	LDH: 274 U/L	-	LDH: 890 U/L	108-212 U/l
	Ferritin: 1,665 ng/mL	-	Ferritin: 1,093 ng/mL	21-267 ng/mL
	Procalcitonin: 0.15 ng/mL	-		<0.06 ng/mL
	CRP: 59 mg/L	-	CRP: 19.1 mg/L	<8.1 mg/dL
	D-dimer: 3,249 mg/mL	-	D-dimer: 53,501 mg/mL	<500 ng/mL

## Discussion

CVD is commonly observed in COVID-19 patients. Analysis of 1,099 COVID-19-infected hospitalized patients identified HTN, DM, and CAD as the most common comorbidities [4]. Patients with preexisting CVD have a higher risk of developing severe disease [5]. Another study analyzing 44,672 COVID-19-infected patients found that the crude fatality rate in patients with CVD was much higher than those without comorbidities [6]. Acute cardiac injury with troponin elevation is one of the common complications in COVID-19-infected patients [7]. With the rapid spread of COVID-19 and the increasing number of deaths, physicians are constantly trying to identify risk factors and mortality-morbidity predictors to triage patients in a timely manner. The main challenge we identified in our cases was the obvious difference in the severity of cardiovascular comorbidities but a similar level of potential myocardial damage from COVID-19. Our patients lacked easily identifiable cardiovascular symptoms such as chest pain. Instead, two patients presented with a more subtle presentation of syncope or presyncope and the third patient presented with classic COVID-19 respiratory symptoms. The postulated pathogenesis of cardiac injury is by viral suppression of ACE2 receptors in the endothelial cells of the vascular system, heart, and lungs, which can lead to injury and direct inflammatory cell infiltration of the myocardium [3,8]. Another theory is the cytokine storm causing multiorgan failure in more critically ill patients. However, our cases highlight that despite the presence of myocardial injury, especially in patients with milder disease presentation, the level of troponin elevation may not predict an associated compromise in cardiac function, as displayed in the difference in their ECG findings. Our cases, however, show that the level of troponin elevation may correlate with overall morbidity and mortality. This raises questions on the diagnostic limitations of troponin elevation in COVID-19 patients and the potential use of ECG in monitoring cardiac output as a reliable way to predict cardiovascular-specific morbidity and mortality. Identifying cardiovascular function early on is important, especially with new literature identifying myocarditis from COVID-19 infection [9,10].

## Conclusions

It is a challenge to identify all the symptomatology related to COVID-19 infection. Cardiac involvement can present with classic symptoms such aschest pain, or less clear respiratory symptoms or syncope, as seen in our patients, making it a challenge for diagnosing cardiac involvement in COVID-19. Further adding to the challenge is the wide variability in the cardiovascular manifestations of COVID-19, including ischemia, lethal arrhythmias, heart failure, and heart blocks. The level of troponin elevation may not predict an associated compromise in cardiac function, thus raising questions on the diagnostic value of troponin elevation in COVID-19 patients. Myocardial injury seems to worsen the prognosis to an uncertain extent. Understanding the cardiac involvement in COVID-19 on a larger scale with future case reports and cross-sectional studies can help better understand these associations and aid physicians to develop high-quality algorithms and better management strategies in approaching COVID-19 patients with cardiovascular involvement.
